# Diseases of the Intestines—Their Special Pathology, Diagnosis and Treatment

**Published:** 1902-01

**Authors:** 


### Books and Magazines.

DISEASES OF THE INTESTINES—Their Special Pathology, Diagnosis and Treatment. With sections on Anatomy and Physiology, Microscopic and Chemic Examination of the Intestinal Contents, Secretions, Feces, and Urine; Intestinal Bacteria and Parasites; Surgery of the Intestines; Dietetics; Diseases of the Rectum, etc. By John C. Hemmeter, M. D., Ph. H., Professor in the Medical Department of the University of Maryland; Consultant to the University and Director of the Clinical Laboratory, etc. In two volumes. Volume I.—Anatomy, Physiology, Intestinal Bacteria, Methods of Diagnosis, Therapy and Materia Medica of Intestinal Diseases, Diarrhea, Constipation, Enteralgia and Enterodynia, Meteorism, Dystrypsia, Enteritis, Colitis, Dysentery, Intestinal Ulcers, Intestinal Neoplasms, etc. With many original illustrations, some of which are in colors. Published by P. Blakiston's Son & Co., 1012 Walnut St., Philadelphia. 1901. Large octavo, 740 pages. Price, $5.00 per volume.

With the possible exception of J. J. Woodward's masterly treatise on diseases of the intestinal tract, published by the United States government, this complete, well illustrated and splendidly written work probably has no equal in the English language. It certainly deserves to rank with Beaumont's "Diseases of the Stomach" and with the best German and French works. It deals with intestinal diseases as they occur in this country, modified by our climate, diet and habits of life, hence is pre-eminently suited to the needs of the American practitioner. From its pages the advanced student of medicine may readily instruct himself concerning the best approved modern methods of diagnosis and treatment of intestinal disorders.

Theoretical discussions and useless elaboration of details have wisely been omitted, an unusual virtue in a work of so great a number of pages. R.

